# Advanced Composite Biomaterials

**DOI:** 10.3390/ma14030625

**Published:** 2021-02-01

**Authors:** Stefan Ioan Voicu, Marian Miculescu

**Affiliations:** 1 Advanced Polymer Materials Group, Faculty of Applied Chemistry and Material Science, University Polytehnica of Bucharest, str. Gheorghe Polizu 1-7, 011061 Bucharest, Romania; 2 Faculty of Applied Chemistry and Materials Science, University Politehnica of Bucharest, Gheorghe Polizu 1-7, 011061 Bucharest, Romania; 3 Faculty of Materials Science, University Politehnica of Bucharest, Splaiul Independentei 313, 060042 Bucharest, Romania; marian.miculescu@upb.ro

“Biomaterials” is one of the most important fields of study in terms of its development in the 21st century. This is due to both the progress of medical science, material science, chemistry, and physics, and the large number of practical needs its scope can address—materials for favoring osteointegration, systems for controlled release of drugs, materials and sites for tissue engineering, etc. From Figure 1, we can see a steady increase in interest in research in the field of biomaterials in the last 10 years, following a simple search of the keyword “biomaterial” across two main databases—Thomson Reuters ISI and Scopus, respectively. The graph shows that, in the evaluated period, approximately 45,000 articles were published, which is well above other areas.

This special number contributes to the vast field of biomaterials presenting both review articles in the field of polymeric biomaterials based on cellulose (hydroxyapatite or graphene composites for tissue engineering), as well as original research articles with various applications, such as controlled release of drugs, composites based on polylactic acid or poly methyl methacrylate. Thus, the controlled release of active pharmaceutical substances by heat generation, under the influence of the stimuli in the magnetic field, can be obtained by the synthesis of implantable polymeric biomaterials, having a practical and crucial contribution in the field of surgery [1]. Propylene-based biomaterials are widely used in abdominal surgery; their mechanical properties and biocompatibility can be improved by obtaining lignocellulose fiber composites [2]. Aerogels have multiple uses in the field of biomaterials; the synthesis of new such systems or the search for materials that are increasingly accessible or with improved properties, being a continuous challenge. The properties of aerogels are crucial for potential applications in the biomedical field, and the improvement of their mechanical and thermal characteristics are reported, including for medical applications that require these properties [3]. One of the most widely used biocompatible polymers is polylactic acid. Hydroxyapatite compounds of this polymer can be successfully used as precursors for 3D printing, as well as for obtaining polymeric biomaterial membranes with potential applications in osteointegration or various separations [4]. The synthesis and characterization of composite films based on polyvinyl alcohol, cellulate nanofibrils and carbon quantum dots have been reported, alongside the potential applications of films obtained in package manufacturing that have special properties (antibacterial, transparent and resistant to ultraviolet [5]). In the field of the optical properties of biomaterials, the capacity and staining mechanism for horn claw (a biocompatible material obtained from biomass) were investigated [6]. The clogging of polymer membranes, especially of separations of viruses or bacteria, is a major problem in the field of the separation of these species. A large study has been reported on the synthesis of silicone rubber composite membranes and graphene oxide with remarkable anti-clogging properties [7]. Moreover, the study showed, in the first place, the influence of the color of the surface of the membrane under conditions of hydrodynamic separation (instead of the conditions of static separation)—the reported data opening a new scientific field. In the field of precursors for implantable dental materials, a new composite based on poly-methyl methacrylate and ZrO_2_ was reported—the composite material proving to have remarkable mechanical properties compared to classical resin [8]. Cellulose derivatives are among the most widely used biocompatible polymers due to the fact that their degradation exclusively releases glucose. For this reason, cellulose-based composites are the most studied and developed precursors at present. Of the many fillers that can be used, graphene (with potential applications in tissue engineering) [9] and hydroxyapatite (especially for osteointegration) [10] are presented in detail in two reviews.

## Figures and Tables

**Figure 1:**
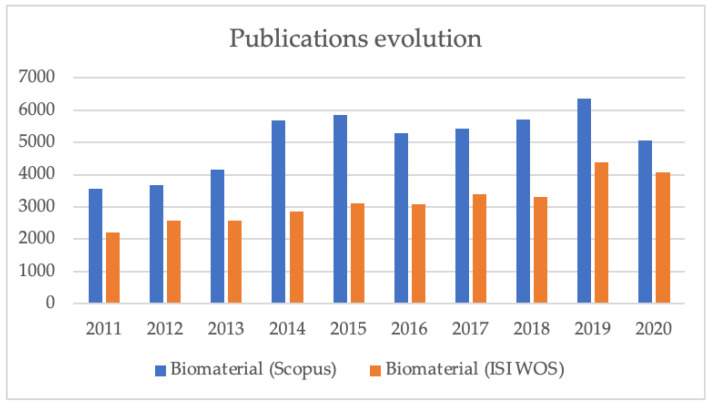
Evolution of the number of publications on Scopus and ISI Web of Knowledge containing “biomaterial” during the period 2011–2020.
